# Supplementary figures and images for: Space-environment relationship in the identification of potential areas of expansion of Trypanosoma cruzi infection in Didelphis aurita in the Atlantic Rainforest
Source: PLoS One. 2023 Jul 28;18(7):e0288595. doi: 10.1371/journal.pone.0288595 (PMC10381050; doi:10.1371/journal.pone.0288595)

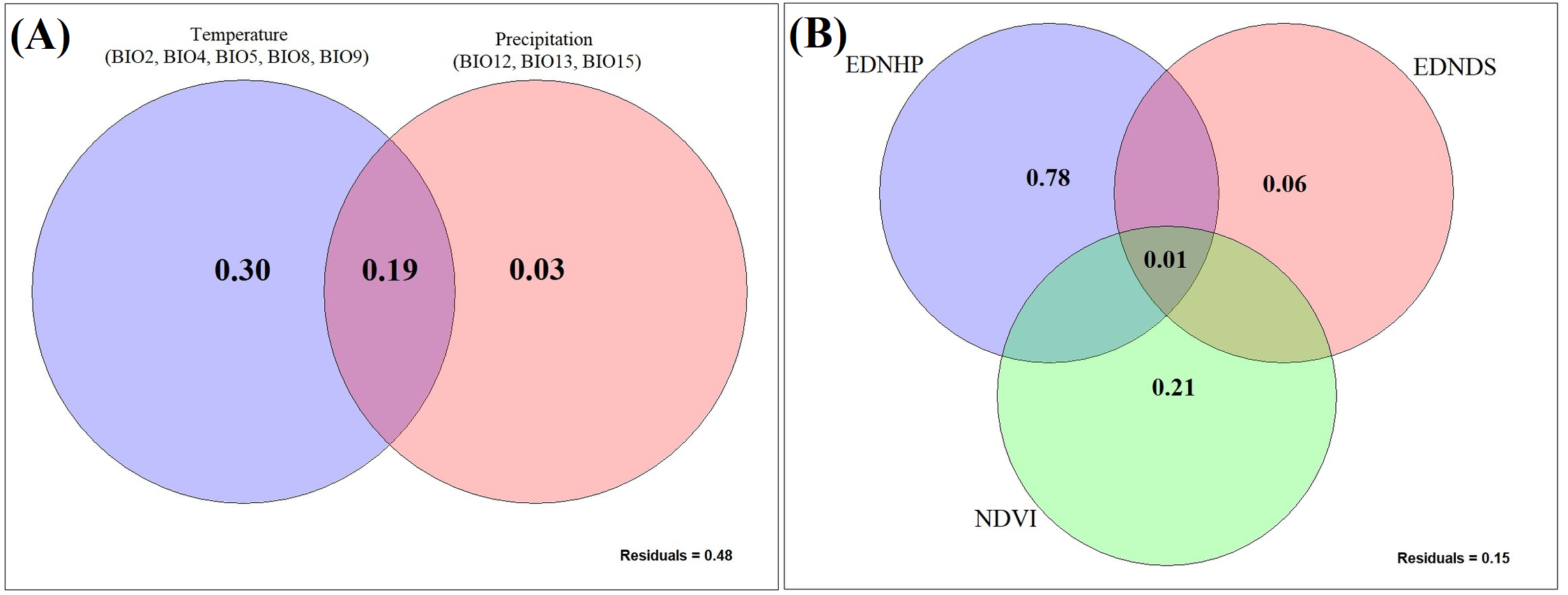

Supplement: S1 Fig — Partition of variance: A) climatic variables of the modeling of Didelphis aurita for the Atlantic Rainforest biome; B) landscape variables of the modeling of Didelphis aurita for the Atlantic Rainforest biome. Standardized data (scale x to zero mean and unit variance), and buffer 0.1°. Values < 0 note shown, and are interpreted as zeros, corresponding to cases where explanatory variables explain less variation than normal random variables [43]. EDNPP: Euclidean Distance to the Nearest Population Presence; EDNDS: Euclidean Distance to the Nearest Drainage Section. Software: RStudio, under R programming language version 4.1.2. (TIF) [file pone.0288595.s001.tif]
