# Supplementary material for: Space-environment relationship in the identification of potential areas of expansion of Trypanosoma cruzi infection in Didelphis aurita in the Atlantic Rainforest
Source: PLoS One. 2023 Jul 28;18(7):e0288595. doi: 10.1371/journal.pone.0288595 (PMC10381050; doi:10.1371/journal.pone.0288595)
Supplement: S1 Appendix — (PDF) [file pone.0288595.s007.pdf]

S1 Appendix. Google Earth Engine code to export the GHS-POP R2015A - GHS population grid, derived from GPW4, multitemporal (1975, 1990, 2000, 2015) to the Atlantic Rainforest area plus 50 km.

```
/*================================
        Beginning of Code
================================== */

/*Selecting the collection of GHS-POP R2015A - GHS population grid, for the period 2010/01/01 to 2015/12/31:*/

var dataset = ee.ImageCollection('JRC/GHSL/P2016/POP_GPW_GLOBE_V1')
                  .filter(ee.Filter.date('2010-01-01', '2015-12-31'));

/*Cutting the image collection for the Atlantic Rainforest area plus 50 km and calculating the median of its pixels:  */
var populationCount = dataset.median().clip(AtlanticRainForest).toInt().select('population_count');

/*Creating a color palette just for viewing images in Google Earth Engine: */
var populationCountVis = {
  min: 0.0,
  max: 200.0,
  palette: ['060606', '337663', '337663', 'ffffff'],
};

/*Plotting the images on the screen: */
Map.setCenter(-42.93, -20.19, 4);
Map.addLayer(populationCount, populationCountVis, 'Population Count');

/*Selecting the spatial resolution information of the images, inserting them in the "scale" field, and exporting them to Google Drive: */
var orig = dataset.first().projection();
var x = populationCount.reproject(orig)
Export.image.toDrive({
  image: populationCount,
  description: 'PopulationCount_AtlanticRainForest',
  scale: x.projection().nominalScale().getInfo(),
  crs: 'EPSG: 4326',
  folder: 'EE',
  maxPixels: 49338893220,
  region: AtlanticRainForest
})

/*================================
          End of Code
================================== */

/*Observation: the definition of the shapefile containing the area of the Atlantic Forest increased
by 50 km is carried out through the "assets" field of Google Earth Engine, so that it is later imported
as a variable and can be used in the code in the ".clip" fields (AtlanticRainForest)" and "region: AtlanticRainForest" */
```
