# Supplementary material for: Space-environment relationship in the identification of potential areas of expansion of Trypanosoma cruzi infection in Didelphis aurita in the Atlantic Rainforest
Source: PLoS One. 2023 Jul 28;18(7):e0288595. doi: 10.1371/journal.pone.0288595 (PMC10381050; doi:10.1371/journal.pone.0288595)
Supplement: Attachment — Submitted filename: Review PONE-D-23-05941_Orozco.docx [file pone.0288595.s009.docx]

Review

Manuscript title: *Space-environment relationship in the identification of potential areas of expansion of Trypanosoma cruzi infection in Didelphis aurita in the Atlantic Rainforest*

This is an interesting and well written manuscript, which combines information on the ecology and epidemiology of *Trypanosoma cruzi* in the Atlantic rainforest, with the use of valuable tools such as Ecological Niche Modeling and the Ecoland approach. This work is well addressed and the models are compelling.

The broad objective of this study was to understand the conditions related to the parasite/host/environment triad in which the transmission of *T. cruzi* occurs involving a generalist wild species (strongly associated with anthropic changes) such as *Didelphis aurita* in the Atlantic Rainforest, a biome whose coverage area has been reduced in recent years, and currently, only 12,4% of its original area is well conserved. The authors hypothesized that the spatial distribution of triatomines *Triatoma*

*vitticeps* and *Panstrongylus megistus*, the host *D. aurita* and their infection by *T. cruzi* is directly related to the physiognomy of the landscape.

Applying ecological niche models, the authors explored the distribution of infection by *D. aurita* and T*. cruzi*, using abiotic and biotic variables, predicting their occurrence. Records of *T. cruzi* infected and non-infected *D. aurita* (detected by hemoculture) were analyzed through climate and landscape approaches by the Ecoland method. The subject is clearly relevant to the journal, the data collected by the authors represent a very impressive and interesting dataset. The results are original and clearly within the scope of the journal. The outcomes could contribute to a better understanding of *T. cruzi* transmission in disturbed environments, where generalist reservoirs such as *D. aurita* are abundant.

I have a concern regarding the use of blood cultures as the only parasitological technique, to determine positivity for *T. cruzi*, without considering the parasite load and infectivity to the vector. I understand that given the time range considered in this work (1992-2019), there are limitations regarding the availability of comparable results and techniques obtained from different databases.

However, given that the high reservoir competence of *Didelphis* genus is known, an interesting point to evaluate is the parasite load in different spatial and environmental contexts, including this variable in the models. Although I understand that this question is outside the scope of this manuscript, in a scenario where environmental changes prevail, there are multiple variables associated with sylvatic cycles and T. cruzi transmission, and the results obtained lead me to wonder about this point. In this study the models showed that areas of high suitability for the presence of marsupials are a necessary, but not sufficient, condition for *D. aurita* to act as a reservoir for *T. cruzi*. I encourage authors to explore, perhaps on a smaller scale, the parasite loads in blood of *D. aurita* as a better approximation to the reservoir competence of this species.

In a changing environmental context, the conclusions of this work are an important management tool in integrated strategies under the One Health approach.

Minor points

Line 64-69: Although hemoculture and blood fresh examination are used, I suggest including xenodiagnosis as a very useful parasitological technique in order to determine the reservoir competence of sylvatic hosts of *T. cruzi.*

Line 819: delete the word funding

Consistent use of abbreviations. For example: use ENM to abbreviate Ecological Niche Modeling, throughout the manuscript (sometimes it appears abbreviated and other times complete).
